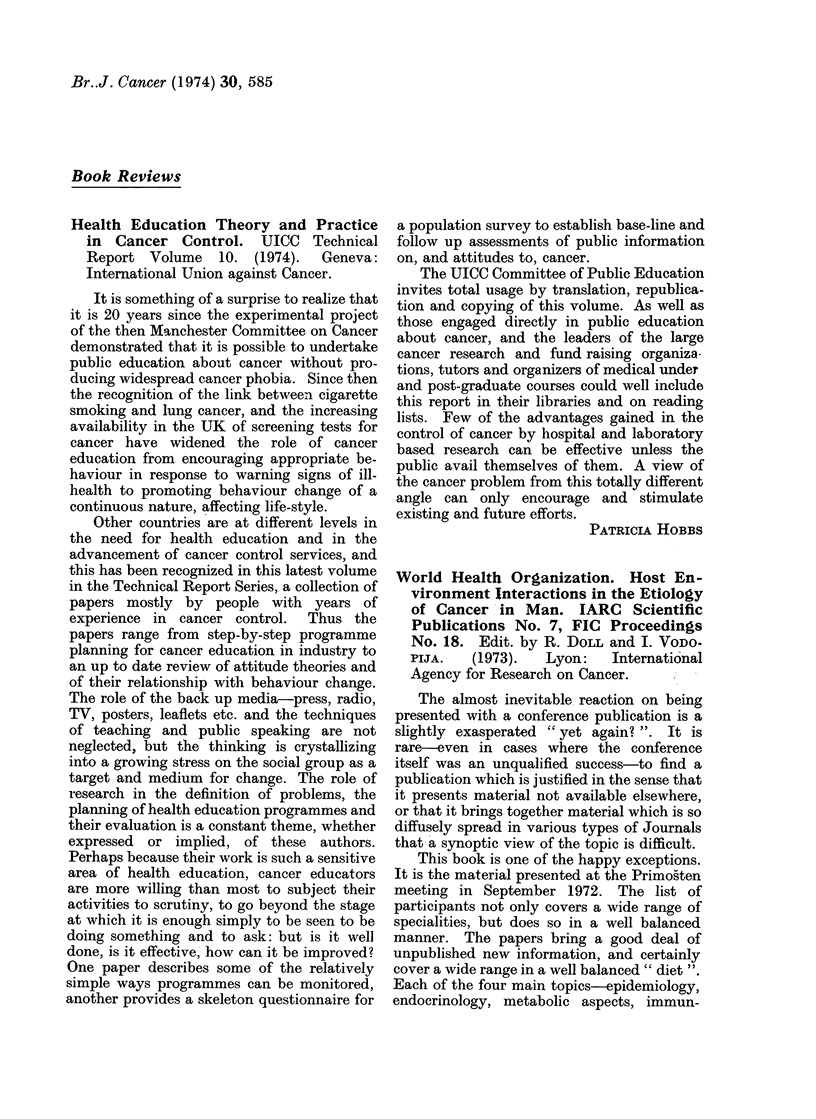# Health Education Theory and Practice in Cancer Control

**Published:** 1974-12

**Authors:** Patricia Hobbs


*Br. J. Cancer* (1974) **30**, 585

### *Book Reviews*

**Health Education Theory and Practice in Cancer Control.** UICC Technical Report Volume 10. (1974). Geneva: International Union against Cancer.

It is something of a surprise to realize that it is 20 years since the experimental project of the then Manchester Committee on Cancer demonstrated that it is possible to undertake public education about cancer without producing widespread cancer phobia. Since then the recognition of the link between cigarette smoking and lung cancer, and the increasing availability in the UK of screening tests for cancer have widened the role of cancer education from encouraging appropriate behaviour in response to warning signs of ill-health to promoting behaviour change of a continuous nature, affecting life-style.

Other countries are at different levels in the need for health education and in the advancement of cancer control services, and this has been recognized in this latest volume in the Technical Report Series, a collection of papers mostly by people with years of experience in cancer control. Thus the papers range from step-by-step programme planning for cancer education in industry to an up to date review of attitude theories and of their relationship with behaviour change. The role of the back up media—press, radio, TV, posters, leaflets etc. and the techniques of teaching and public speaking are not neglected, but the thinking is crystallizing into a growing stress on the social group as a target and medium for change. The role of research in the definition of problems, the planning of health education programmes and their evaluation is a constant theme, whether expressed or implied, of these authors. Perhaps because their work is such a sensitive area of health education, cancer educators are more willing than most to subject their activities to scrutiny, to go beyond the stage at which it is enough simply to be seen to be doing something and to ask: but is it well done, is it effective, how can it be improved? One paper describes some of the relatively simple ways programmes can be monitored, another provides a skeleton questionnaire for a population survey to establish base-line and follow up assessments of public information on, and attitudes to, cancer.

The UICC Committee of Public Education invites total usage by translation, republication and copying of this volume. As well as those engaged directly in public education about cancer, and the leaders of the large cancer research and fund raising organizations, tutors and organizers of medical under and post-graduate courses could well include this report in their libraries and on reading lists. Few of the advantages gained in the control of cancer by hospital and laboratory based research can be effective unless the public avail themselves of them. A view of the cancer problem from this totally different angle can only encourage and stimulate existing and future efforts.

PATRICIA HOBBS